# A Real Time *Metridia* Luciferase Based Non-Invasive Reporter Assay of Mammalian Cell Viability and Cytotoxicity via the β-actin Promoter and Enhancer

**DOI:** 10.1371/journal.pone.0036535

**Published:** 2012-05-09

**Authors:** Shawn E. Lupold, Tamara Johnson, Wasim H. Chowdhury, Ronald Rodriguez

**Affiliations:** The James Buchanan Brady Urological Institute, Johns Hopkins University School of Medicine, Baltimore, Maryland, United States of America; University of Central Florida, United States of America

## Abstract

Secreted reporter molecules offer a means to evaluate biological processes in real time without the need to sacrifice samples at pre-determined endpoints. Here we have adapted the secreted bioluminescent reporter gene, *Metridia* luciferase, for use in a real-time viability assay for mammalian cells. The coding region of the marine copepod gene has been codon optimized for expression in human cells (hMLuc) and placed under the control of the human β-actin promoter and enhancer. *Metridia* luciferase activity of stably transfected cell models corresponded linearly with cell number over a 4-log dynamic range, detecting as few as 40 cells. When compared to standard endpoint viability assays, which measure the mitochondrial dehydrogenase reduction of tetrazolium salts, the hMLuc viability assay had a broader linear range of detection, was applicable to large tissue culture vessels, and allowed the same sample to be repeatedly measured over several days. Additional studies confirmed that MLuc activity was inhibited by serum, but demonstrated that assay activity remained linear and was measurable in the serum of mice bearing subcutaneous hMLuc-expressing tumors. In summary, these comparative studies demonstrate the value of humanized *Metridia* luciferase as an inexpensive and non-invasive method for analyzing viable cell number, growth, tumor volume, and therapeutic response in real time.

## Introduction

High throughput cell viability and cytotoxicity assays are a mainstay for the biologic and therapeutic community. Standard cell viability assays quantify metabolically active cells through dehydrogenase activity, which converts tetrazolium salts into measurable colorimetric products [1], [2]. Cell proliferation can also be quantified by genomic incorporation of radiolabeled Thymidine or 5-bromo-2-deoxyuridine [3]. These standard viability and cell proliferation assays require cell lysis and therefore a predetermined and optimized endpoint for each specific condition; plus additional materials and steps for signal quantification. The cost of these assays grows with the evaluation of temporal events, where separate samples and controls are requiredre of these assays grow with the ps for signal quantificationn of radiolabeled thymidine ime basis and overcome the need for r for each measured time point. Cytotoxicity assays can overcome the need for cell lysis by measuring leaky dehydrogenase activity in the supernatant, which escapes through the damaged membranes of dead or dying cells [4], [5]. However, the sensitivity of these assays is generally limited to over 1,000 cells per multiwell plate and the dynamic range of spectrophotometers. Given the limitations of these cell viability and cytotoxicity assays, the application of stably expressed reporters becomes practical for commonly used cell lines and tumor models [6].

Secreted reporters provide a means to evaluate biologic events in real time, thus allowing a flexible endpoint and overcoming the need for multiple replicates of plates in time course studies. Recently a naturally secreted luciferase was identified and cloned from the marine copepod *Metridia longa* [7]. This 23 kilodalton enzyme, *Metridia* Luciferase (MLuc), is attractive as a reporter because it catalyzes a simple bioluminescent reaction which only requires coelenterazine and oxygen. The product is a blue bioluminescent signal (λ_max_ = 480 nm) which is detected with high sensitivity over a broad dynamic range in conventional luminometers [7]. Since this discovery additional secreted luciferase genes have been cloned, including MpLuc1 and MpLuc2 from the related *Metridia pacifica* copepod and *Gaussia* luciferase (GLuc) from the *Gaussia princeps* copepod [8], [9]. All of these secreted proteins have great promise as reporters for high throughput and non-endpoint monitoring of biologic processes.

Here we have applied codon optimization of the reported MLuc coding region [7] to generate a humanized version of *Metridia* Luciferase, hMLuc. We then created a mammalian cell viability reporter by placing the hMLuc gene downstream of the constitutively expressed human β-actin promoter and enhancer, incorporating the native first intron of the β-actin gene to facilitate high level expression and efficient processing. The reporter system was assessed in human and mouse cancer and cell line models. The results support that the assay is inexpensive, highly sensitive, linear over a range of several logs, and applicable to a variety of cell culture vessels. In addition, we studied the application of β-actin driven hMLuc as an *ex vivo* reporter in tumor bearing mouse models. The results confirm that serum does inhibit MLuc activity but, contrary to previous reports [10], we reveal that hMLuc can be detected as an *ex vivo* reporter in mouse tumor model systems.

## Results

### *Metridia* Luciferase Viability Vector

**Figure 1:**
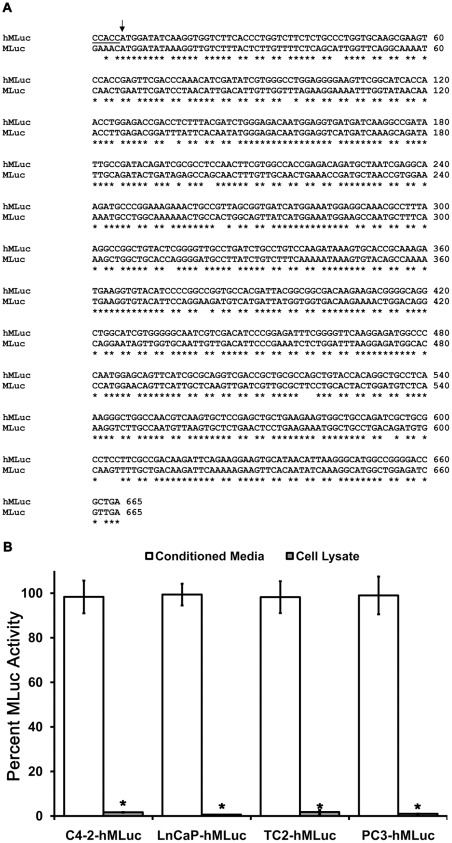
Humanized *Metridia* Luciferase and its cellular partitioning. (A) Sequence Alignment of the codon optimized *Metridia* Luciferase (hMLuc) and the *Metridia longa* Luciferase (MLuc). The engineered Kozak sequence is underlined and the arrow points to the start codons. matches are indicated by (*) below. Overall similarity of 75.3%. (B) MLuc is secreted from human cells. Various clones of stably expressing hMLuc prostate cancer cell lines were evaluated for MLuc activity from conditioned medium and cell lysates. The percent value of total MLuc activity per clone is reported. Error bars represent standard error of the mean from 12 samples. *P<0.05 relative to Media, (t-student test).

The human β-actin gene is one of the most highly active genes in non-muscle mammalian cells and is a standard reference gene for many biological assays. promoter for β-actin is well characterized and consists of a core promoter and downstream enhancer, which resides in the first intron [11], [12]. strong promoter/enhancer was applied to generate constitutive expression of the secreted *Metridia longa* luciferase reporter. To ensure optimal expression and translation, the MLuc coding region was humanized by codon optimization and engineered with a Kozak consensus sequence and SV40 polyadenylation signal (Figure 1A). The humanized coding sequence, hMLuc, has 75.3% sequence identity with the copepod MLuc. The vector was designed to produce a spliced transcript, where the β-actin intron 1 and enhancer are removed, leaving only the coding region of the bioluminescent reporter.

The resulting reporter vector, pDonor-hβ-Actin-hMLuc, was used to generate stable transfectants in several human (C4-2, LNCaP, PC-3, HCT-116) and mouse (Tramp-C2) cell lines. blasticidin selection and clonal isolation, individual clones were evaluated for MLuc activity in cellular lysates and in the conditioned media. Figure 1B reveals that the majority of the MLuc activity was detected in the conditioned media, with little to no detectible activity in the cell lysate. This demonstrates that the humanized MLuc is efficiently transcribed, translated, and that nearly 100% of the reporter is secreted into the media of transfected mouse and human cell models.

### Correlation of reporter activity with cell number

**Figure 2:**
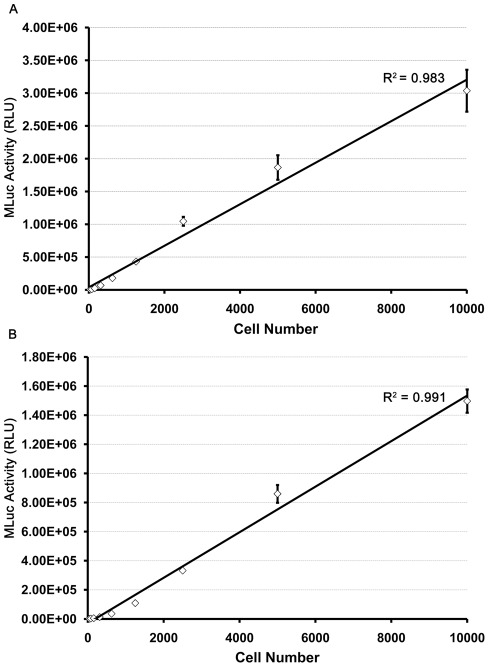
Linear quantification of MLuc Activity with cell number. Serially diluted samples of (A) TC-2-hMLuc and (B) LNCaP-hMLuc were plated in 96 well plates. Twenty four hours after plating, 100 microliters of conditioned media was taken from each well and evaluated for MLuc activity. Error bars represent standard error of the mean from 8 samples. The X-axis represents plated cell number and the Y-axis represents MLuc Activity (Relative Light Units or RLU) for each sample. Linear regression analysis indicates an R^2^ value of 0.983 for (A) TC-2-hMLuc cells and 0.991 for (B) LNCaP-hMLuc cells.

Two cell lines, LNCaP-hMLuc and TC2-hMLuc, were serially diluted into multiwell tissue culture plates to determine the correlation of MLuc activity to plated cell number. MLuc activity was detectable in cell culture media containing as few as 40 cells and the MLuc activity correlated linearly with cell number to the highest concentration of cells tested (Figure 2A & B). A correlation coefficient of 0.991 and 0.983 were obtained over a 4-log range for LNCaP-hMLuc and TC2-hMLuc, respectively. Further studies with stable HCT-116-hMLuc colon cancer cells and transiently transfected HEK293 human embryonic kidney cells support the sensitivity and linearity of this reporter assay (Figure S1; Methods S1). This indicates that β-actin-driven hMLuc activity may be a robust and highly sensitive real time assay for viable cells number.

### hMLuc Viability Assays

**Figure 3:**
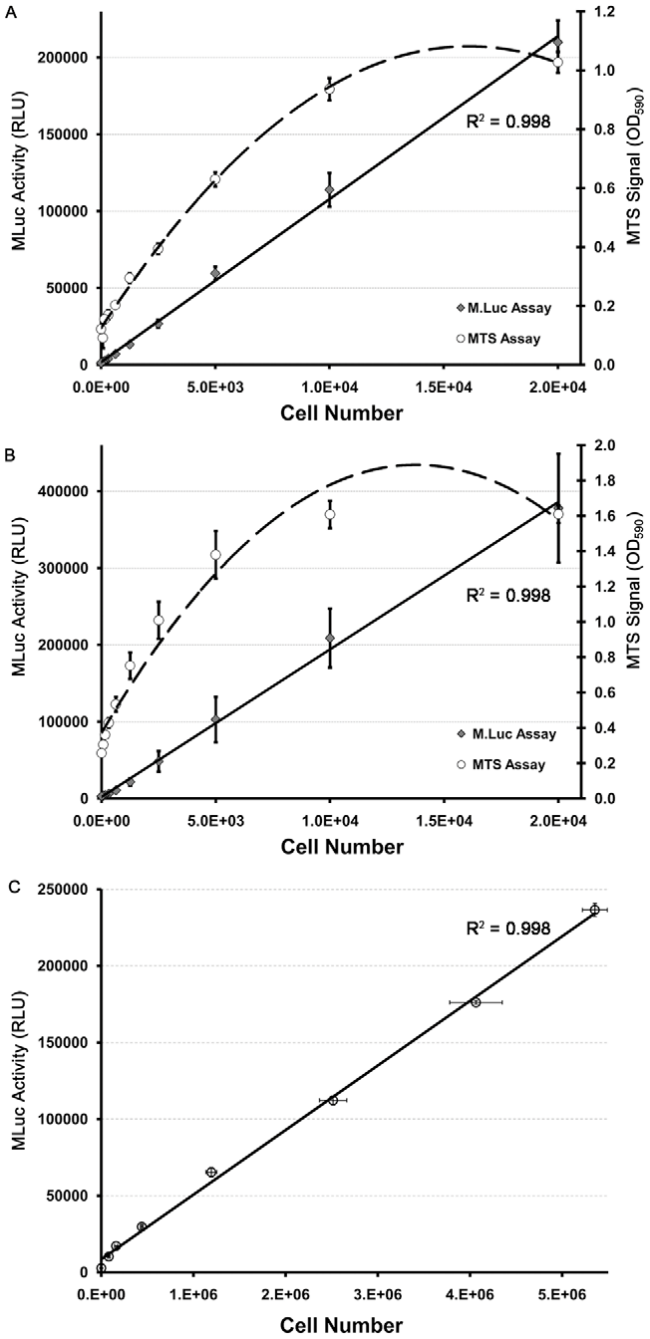
Comparison of MLuc and MTS viability Assays. Serially diluted samples of (A) TC-2-hMLuc and (B) LNCaP-hMLuc were plated in 96 well plates. Twenty four hours after plating cell supernatants were evaluated for MLuc Activity and the remaining cells by MTS viability assay. The Y-Axis to the left quantifies MLuc activity (RLU) where the Y-Axis to the right quantifies MTS signal (absorbance of 590 nm light after four hours of incubation with MTS reagents). The X-axis represents plated cell number. N = 8. Error bars represent standard error of the mean. Trendlines were fitted for linear regression (MLuc) or Power curves (MTS) over the given range: (A) TC-2-hMLuc linear regression, R^2^ = 0.998. (B) LNCaP-hMLuc linear regression R^2^ = 0.998. (C) Serially diluted LNCaP-hMLuc were plated in 25 cm^2^ culture flasks (N = 2). Twenty four hours after plating cell supernatants were evaluated for MLuc Activity and the remaining cells were counted by trypan blue exclusions. The Y-axis represents MLuc Activity (RLU) and vertical error bars represent standard error between samples. The X-axis represents counted viable cell and horizontal error bars represent standard error between samples. Linear regression, R^2^ = 0.998.

To directly compare the hMLuc viability assay to standard tetrazolium salt based viability assays, LNCaP-hMLuc and TC2-hMLuc cells were serially diluted over a range of 4-logs and plated on standard 96-well tissue culture plates. Twenty four hours after plating, cell viability of the same sample was evaluated by both 5-(3-carboxymethoxyphenyl)-2-(4,5-dimethylthiazoly)-3-(4-sulfophenyl)tetrazolium, inner salt (MTS) reduction, and by hMLuc Viability Assay. Specifically, a fraction of the conditioned media was acquired from each plate and quantified for MLuc activity, while the remaining cells were quantified by MTS assay. Figure 3A demonstrates that MLuc activity correlated linearly with TC-2-hMLuc cell number (R^2^ = 0.998) over the full dilution range. The MTS Assay signal was similarly linear for a portion of the dilution, but lost linearity at the dilution extremes. When compared to MLuc, the MTS assay did not show as broad a dynamic range as the bioluminescent reporter assay. Similarly, in Figure 3B, LNCaP-hMLuc cell number correlated linearly with MLuc activity over the full dilution range tested (R^2^ = 0.998), whereas the linear range of the MTS assay was limited to approximately 5,000 cells. It is important to note that these results do not indicate that the MTS assay is not functional over a broad range of cell densities; it merely demonstrates a limitation to the dynamic range of MTS in a single assay. MTS substrate concentration and incubation time may be modified for optimum linearity in a lower or higher cells density samples; however, this will require multiple plates and optimization at each cell density extreme.

Another limitation of standard viability assays is the format in which it can be applied. For example, it would be difficult and expensive to quantify the number of viable cells in a large tissue culture flask or roller bottle by MTS reduction. Here we assessed the accuracy of secreted hMLuc to quantify cell number in larger tissue culture vessels by serially diluting LNCaP-hMLuc cells into 25 cm^2^ screw top tissue culture flasks. Media and cells were then harvested after 24 hours. Viable cell numbers were counted on a standard hemocytometer by trypan blue exclusion and correlative hMLuc levels were measured by MLuc assay. The results confirm a direct linear correlation (R^2^ = 0.998) of MLuc activity with viable cell number (Figure 3C). Thus, the hMLuc viability assay could be applied to assess long term cell growth and viability in a variety of cell vessels.

### Real time quantification of cell growth and therapeutic effect

We next applied the hMLuc viability assay to follow cell growth and therapeutic effect in an androgen receptor (AR) targeted therapeutic model. Both LNCaP and TC-2 cell lines are AR positive and sensitive to androgen deprivation therapy by the steroidal anti-androgen Bicalutamide.

**Figure 4:**
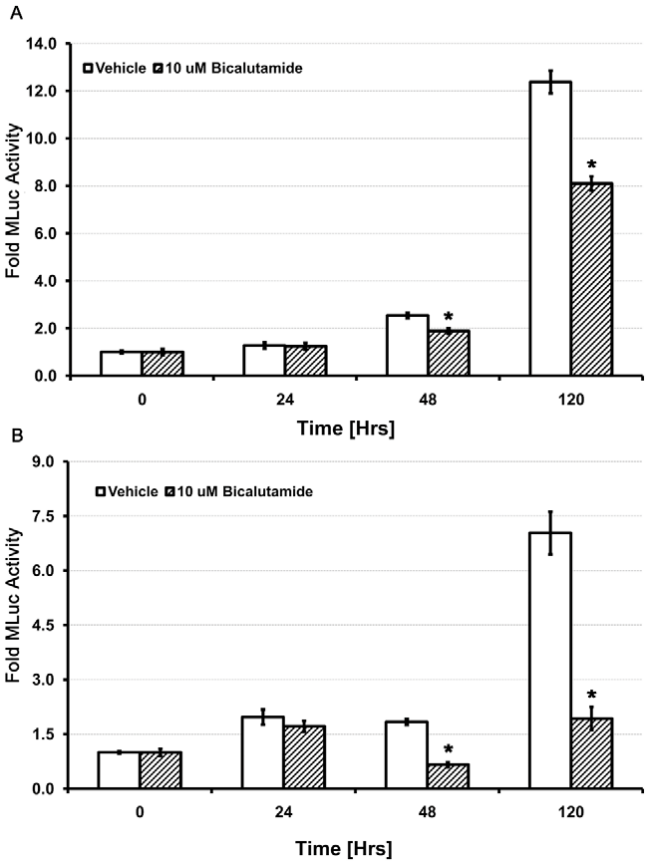
Evaluation of growth and therapeutic effect by MLuc Viability Assay. (A) TC2-hMLuc cells and (B) LNCaP-MLuc cells were treated with a 10 micromolar dose of the anti-androgen Bicalutamide (Casodex) or vehicle and viable cell numbers over a given time were evaluated by MTS Assays (N = 8). Y-Axis represents fold MLuc activity relative to time zero. Statistical significance was determine by student'st-test (*p<0.05). Error bars represent the standard error of the mean.

TC-2-hMLuc and LNCaP-hMLuc cells were grown for several days in the presence or absence of 10 µM Casodex (Bicalutamide). Cell viability and therapeutic effect were assessed continuously over a five day period by measuring aliquots of cellular media for secreted MLuc activity. The results demonstrate cell growth over time in both controls; with a significant reduction in growth of the Bicalutamide treated cells at the last measured time point (Figure 4). The therapeutic effect was confirmed by MTS assay. This study reveals the benefits of real time measurements (growth and therapeutic effect) in a single vessel without the need to first determine an optimized endpoint.

### Secreted MLuc as an *ex vivo* Reporter Molecule

**Figure 5:**
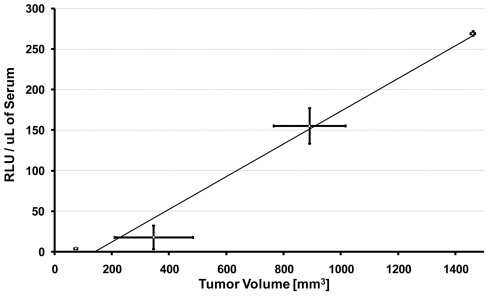
*Ex vivo* quantification of MLuc activity. LNCaP-hMLuc cells were subcutaneously established in a group of four nude mice. Once palpable tumors were established, tumor volume was measured approximately every ten days over several weeks. Blood was simultaneously drawn at the time of measurement for reference MLuc activity. The data was grouped into different tumor volume groups. In all cases, the average serum MLuc activity correlated well with the average tumor volume (R^2^ = 0.981). Error bars represent standard error of the mean for each measurement per group.

**Figure 6:**
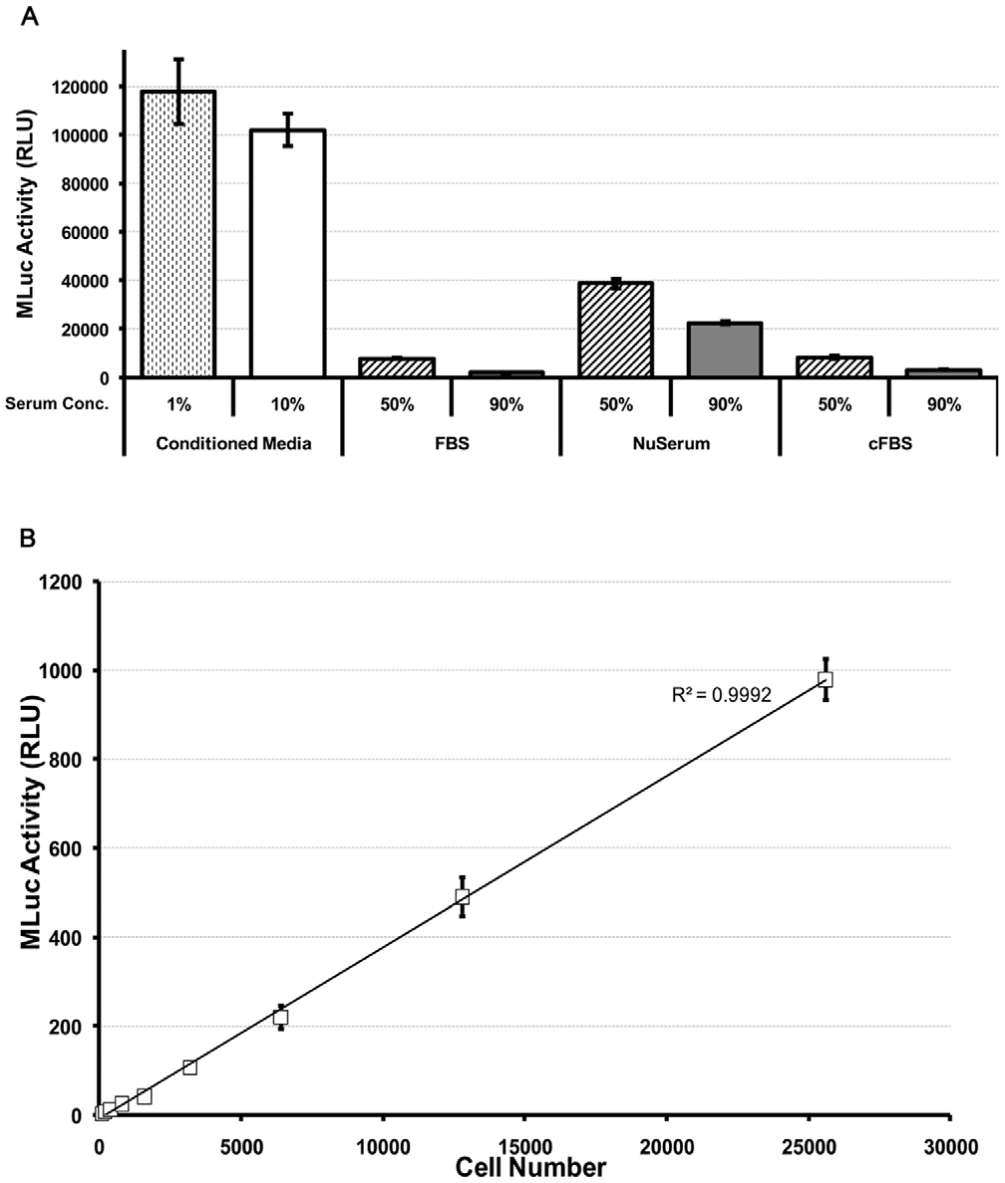
Effect of Serum on MLuc Activity. MLuc conditioned media from TC2-hMLuc was spiked with varying amounts of different serum and bioluminescence activity was quantified. (A) Various serum used in standard culture medium demonstrated a dose dependent quenching of MLuc activity. (B) Despite the quenching, the hMLuc viability assay correlated linearly with TC-2-hMLuc cell number over a broad dynamic range in the presence of ∼80% serum. Error bars represent the standard error of the mean. N = 8. R^2^ = 0.9894.

We completed a pilot study to determine if MLuc activity was quantifiable *ex vivo,* in the blood of mice bearing subcutaneous LNCaP-hMLuc tumors. Blood was collected via tail vein bleed at the time of tumor measurement and the MLuc activity measured in the serum over a period of several weeks. The ratio of tumor volume to MLuc activity per microliter of serum was combined from several animals and time points to determine their correlation. Our results indicate that MLuc activity correlated linearly with measurable tumor volume (Figure 5). These results were surprising because it had been previously reported that MLuc activity was undetectable in rat serum and urine [10]. In that study, Hiramatsu and colleagues identified serum albumin as a strong inhibitor of MLuc activity. We therefore completed similar studies and confirmed that all types of serum tested quenched hMLuc activity in a dose dependent manner; nonetheless, the signals were above background (Figure 6A). Even at high serum concentrations, the dynamic range of the assay was retained and MLuc activity correlated linearly with cell number (Figure 6B). Therefore we confirm that MLuc activity is sensitive to serum inhibition, but that it retains sufficient activity to be applicable as an *ex vivo* serum reporter in nude mouse tumor models.

## Discussion

Bioluminescent reporters provide a method to quantify biologic processes in real time. From firefly luciferase glowing tumors to high throughput pharmaceutical screens, the strong signal and broad dynamic detection range have made bioluminescence invaluable. Secreted bioluminescent reporters, such as MLuc and GLuc, provide new means to quantify biological processes without the need to harvest or lyse cells. Importantly, real time assessment saves reagents, time, and overcomes the need for a predetermined endpoint. While bioluminescent activity of non-secreted reporters can be quantified in intact cells, such as with *in vivo* bioluminescent imaging, the reaction is reliant upon efficient diffusion and retention of the substrate. It is notable that some bioluminescent substrates, such as firefly luciferin, can be substrates for multidrug resistant pumps and therefore partitioned away from the reporter enzyme [13]. In addition, real time luciferase imaging can be affected by the biodistribution of the substrate following intravenous or intraperitoneal injection [14]. Therefore, secreted biolouminescent reporters offer a new mechanism to overcome some of the limitations associated with cytoplasmic bioluminescent reporters.

While secreted bioluminescent reporters are relatively new, they have been successfully applied to a variety or studies. MLuc has been applied to evaluate biological signaling processes *in vitro* [7], where GLuc has been used for *in vivo* imaging [9], *ex vivo* quantification of tumor bourdon [15], and cell viability in drug screens using CMV-promoter driven expression [16]. Here we provide new evidence that hMLuc, when combined with the β-actin promoter/enhancer, can efficiently quantify cell viability and cytotoxicity over a broad dynamic range. Human β-actin is constitutively expressed at high levels in most cell types and is a well accepted reference gene or protein in most biologic assays [17]. However, it is know that β-actin is not a suitable internal control for every assay [18]; therefore, it may not be applicable to every mammalian cell type or growth condition. In our studies, β-actin promoter activity correlated with cell number over a broad range and proved to be a sensitive reporter for detecting cell number *in vitro* in a variety of human and mouse cell lines, different culture vessels, and experimental conditions.

Currently, there are three commonly applied secreted luciferase reporters: MLuc, GLuc, and *Cypridina* Luciferase (CLuc) [19]. The primary advantage of MLuc, versus GLuc and CLuc, may be in the efficiency of secretion. In a direct comparison of these three reporters in HEK293 cells, only 0.86% of hMLuc was retained in the cells, compared to 2.97% and 7.10% for GLuc and CLuc, respectively (Figure S2A; Methods S1). Therefore, MLuc may be more effectively combined with other non-secreted luciferase enzyme reporters, such as Firefly Luciferase and Renilla Luciferase (RLuc), in multi-reporter assays. On the other hand, the advantages of GLuc and CLuc reporters may be in their quantum yield (Figure S2B). GLuc and CLuc demonstrated 2.6 and 4.7 fold greater activity, respectively, than MLuc in this study. Other differences between these three reporters include size (∼20 kD for MLuc and GLuc, versus 61 kD for CLuc) and pH sensitivity. In addition, modifications of these reporters can dramatically affect activity. For example, amino truncation of the MLuc enzyme resulted in greater bioluminescent activity [20].

Recently Hiramatsu and colleagues published a study which directly compared the activity of MLuc and Secreted alkaline phosphatase (SEAP) as *ex vivo* reporters [10]. The study demonstrated that SEAP was a highly sensitive *ex vivo* reporter, whereas MLuc activity was undetectable in the serum or urine of the applied rat model. Moreover, they revealed the MLuc activity was significantly inhibited by serum albumin. Here we confirm that MLuc activity is significantly repressed by serum proteins; however, we found that MLuc activity was still detectable in high serum concentrations and that hMLuc activity retained linearity over the same dynamic range. These results indicate that MLuc is applicable as an *ex vivo* reporter; however, its full potential is limited by serum. Nonetheless, we found that hMLuc was detectable as an *ex vivo* reporter in nude mice bearing subcutaneous LNCaP-hMLuc tumors. The level of hMLuc activity correlated with tumor volume in this small study set (Figure 5). Several factors may have resulted in the greater level of MLuc detection over the previous study, including the application of different promoters (CMV versus β-actin promoter/enhancer), animal models (Sprague-Dawley rat versus nude mouse), cellular location (intraperitoneal versus subcutaneous), codon usage (native versus humanized), or reporter and cell dose (mesangial cells versus established tumors).

In summary, these studies validate secreted *Metridia* Luciferase as a noninvasive reporter for mammalian cell and tumor models. Through the use of the β-actin promoter and enhancer, hMLuc expression was detected with high sensitivity and correlated with over a broad linear range with viable cell number. This *in vitro* viability assay system provides a new mechanism to assess cell viability and cytotoxicity in real time. Finally these studies reveal that hMLuc is applicable as an *ex vivo* reporter, but that detection can be limited by serum inhibition.

## Materials and Methods

### Vectors and Cloning

A custom expression vector, pDonor, was generated from pCMV/Bsd. A Lox71 recombination site was subcloned into BamHI/HindIII sites using linkers (5′-GATCTACCGTTCGTATAGCATACATTATACGAAGTTAT and 3′-AGCTATAACTTCGTATAATGTATGCTATACGAACGGTA), followed by a second Loxm2/66 site inserted into NheI/Apa sites using linkers (5′- ATAACTTCGTATATGGTTTCTTATACGAACGGTA and CTAGTACCGTTCGTATAAGAAACCATATACGAAGTTATGGCC). These lox site offer a means for unidirectional cre/lox recombination [21], [22]; although they were not applied to this project. The *Metridia longa* luciferase gene was codon optimized for human expression (referred to herein as hMLuc) using Vector NTI software (Invitrogen Corporation, Carlsbad, CA) and custom synthesized, with Kozak sequence and SV40 polyadenylation signal (Bioclon Inc. San Diego, CA), and subcloned into the EcoRI/PacI site of pDonor, generating pDonor-hMLuc. A small fragment of hMLuc was subcloned into pGL3 using NheI/BsrGI. The human β-actin promoter and intron-1-enhancer was PCR amplified from genomic DNA (Primers: R0444: AGACCCAGGCTGTGTAGACCCA and R0445 CATCATCCATGGTGAGCTGCG) and the resulting PCR product was subcloned upstream of hMLuc fragments using NheI/NcoI, generating pGL3-Temp2. The promoter and hMLuc fragment were then subcloned back into pDonor-hMLuc using NheI/BsrGI, replacing the CMV promoter, and generating pDonor-hβ-Actin-hMLuc.

### Cell Culture

LNCaP, C4–2, and PC-3 human prostate cancer cells and HEK293 human embryonic kidney cells were obtained from ATCC. HCT116 colon cancer cells were obtained from Bert Vogelstein (Johns Hopkins). Tramp-C2 murine prostate cancer cells were obtained from Norman Greenberg (Baylor College of Medicine). Stable pDonor- hβ-Actin-hMLuc transfectants were established through Blasticidin selection (Invitrogen Corporation). Clones were isolated by limited dilution and confirmed by MLuc assay and genomic PCR. LNCaP-hMLuc, C4-2-hMLuc, and PC3-hMLuc were grown in RPMI 1640 containing 10% FBS and 5 ug/mL Blasticidin. Tramp-C2-hMLuc (referred to herein as TC2-hMLuc) was grown in DMEM containing 5% FBS, 5% NuSerum, 5 ug/mL Blasticidin, 5 µg/mL Insulin, and 0.01 nM R1881.

### Analysis of cellular fractions

1×10^6^ TC2-hMLuc, LNCaP-hMLuc, C4-2-hMLuc, and PC3-hMLuc cells in 100 µl of media were plated (minimum of quadruplets) in 96 well solid white flat bottom polystyrene microplates (Corning, Cat#3917, Lowell, MA). After 24 hours, conditioned media was harvested and remaining cells were lysed with 20 µl passive lysis buffer (Promega, Cat#E194A, Madison, WI). Media and cell lysate were assayed for MLuc activity using 100 µl of a previously described Renilla buffer [23], consisting of 1.1 M NaCl, 2.2 mM Na_2_EDTA, 0.22 M K_x_PO_4_ (pH 5.1), 0.44 mg/mL BSA, 1.3 mM NaN_3_, 1.43 µM coelenterazine, with the final pH = 5.0. Assays were read in a Perkin Elmer Micro Beta luminometer.

### Cellular dilution studies

1×10^4^ TC2-hMLuc and LNCaP-hMLuc cells were serially diluted 2-fold (10,000–39 cells) and plated in a minimum of quadruplets in Falcon 96 multiwell plates. After 24 hours 100 µl of the conditioned media was transferred to a Corning multiwell plate and MLuc activity measured as described above. For larger vessel studies, LNCaP-hMLuc cells were diluted in 25 cm^2^ culture flasks (2.5×10^6^−1×10^6^ cells). After 24 hours 100 µl of the conditioned media was transferred to a Corning multiwall plate for MLuc quantification. The remaining cells were harvested by tryspinization and viable cells counted by trypan blue exclusion. Correlation coefficients were determined by linear regression analysis.

### MTS and β-actin-hMLuc viability assay comparisons

A 2-fold serial dilution of TC2-hMLuc and LnCaP-hMLuc cells were plated (minimum of quadruplet) in Falcon 96 well tissue culture plates (20,000–78 cells). After 24 hours half of the media (100 µl) was transferred to a Corning multiwell plate for MLuc Activity assessment. Standard MTS assays (4 hr development) were performed according to manufacturer′s protocol (Promega CellTiter96 Non radioactive Cell Proliferation Assay) on the cells remaining on the plates. Best line fit for MTS assays were obtained by generating polynomial trendlines while linear regression was applied for MLuc.

### Assay of growth and therapeutic response

500 TC2-hMLuc cells and 1250 LNCaP-hMLuc cells were plated in two Falcon 96 well tissue culture plates. 24 hrs after plating samples were quantified by MTS or MLuc (as described above) to obtain a baseline value. Samples were treated with vehicle or 10 µM Casodex (Bicalutamide, LKT Laboratories, St Paul, MN) for the duration of the experiment. Media (50–100 µl) was collected at 24, 48 & 120 hr intervals for MLuc activity. In time course assays, cell media was refreshed 24 hours prior to each time point to establish a fresh background. At 120 hrs a standard MTS assays was performed.

### *In Vivo* Studies

Studies were performed according to protocols approved by the Animal Care and Use Committee at Johns Hopkins University. Subcutaneous tumors were established in male nude mice (*nu/nu*, Harlan Laboratories, Inc) with 200 µl of 1∶1 matrigel:PBS containing 1×10^6^ LnCaP-hMLuc cells. Once measurable, the tumors and serum MLuc activity were measured weekly. Two healthy mice were used for background references. In brief, 100 µl of blood was collected into a serum tube (Sarstedt, Cat# Microvette 200Z, Newton, NC) via tail vein bleed and tumor volumes were measured (volume  =  L x H x W) approximately every 10 days. 5 µl of serum was diluted into 100 µl of DMEM media & 100 µl Renilla buffer was added to measure MLuc activity. The data from four mice were pooled and plotted to determine the correlation between tumor size and MLuc activity.

### Serum Inhibition Assays

MLuc conditioned media was spiked with FBS, cFBS or NuSerum to a final concentration of 50% or 90%. MLuc Activity was then measured as described above. To determine assay linearity, LnCaP-hMLuc cell dilutions were plated on multiwell plates. The next day 20 µl media from each well was transferred to a Corning multiwell plate & spiked with 80 µl FBS. 100 µl Renilla buffer was added to measure the MLuc activity.

## Supporting Information

Methods S1 Supplementary Materials and Methods.
(PDF)

Figure S1 **Linear quantification of MLuc Activity with cell number.** Serially diluted samples of (A) stable HCT116-hMLuc and (B) transiently transfected HEK293 cells (pDonor-hβactin-hMLuc) were plated in 96 well plates (n = 5). Twenty four hours after plating, 100 microliters of conditioned media was taken from each well and evaluated for MLuc activity. Error bars represent standard error of the mean. The X-axis represents plated cell number and the Y-axis represents MLuc Activity (Relative Light Units or RLU) for each sample. Linear regression analysis indicates an R^2^ value of 0.998 for (A) HCT116-hMLuc cells and 0.987 for (B) HEK293 cells.
(PDF)

Figure S2 **Comparison of secreted luciferase constructs.** HEK293 cells were transiently transfected with RpF-GFP and pCDNA-3.1-hMLuc, pCDNA-3.1-GLuc, or pCDNA-3.1-CLuc. Forty eight hours after transfection luciferase activity was measured from conditioned medium and cell lysates and RLU activity normalized to GFP expression. the (A) percent activity of cell fractions as well as the (B) total activity as reported as Relative Light Units (RLU) are plotted. Error bars represent standard error of the mean. N = 8.
(PDF)
