# Supplementary material for: A Real Time Metridia Luciferase Based Non-Invasive Reporter Assay of Mammalian Cell Viability and Cytotoxicity via the β-actin Promoter and Enhancer
Source: PLoS One. 2012 May 9;7(5):e36535. doi: 10.1371/journal.pone.0036535 (PMC3348918; doi:10.1371/journal.pone.0036535)
Supplement: Methods S1 — Supplementary Materials and Methods. (PDF) [file pone.0036535.s001.pdf]

SUPPLEMENTARY MATERIALS AND METHODS

Cell lines and Materials. HEK293 cells were obtained from ATCC and maintained in DMEM + 10% FBS. Stable pDonor- hβ-Actin-hMLuc transfectants were established through Blasticidin selection (Invitrogen Corporation). HCT-116-hMLuc clones were isolated by limited dilution and confirmed by MLuc assay and genomic PCR. HCT-116-hMLuc were grown in McCoy's 5A media containing 10% FBS and 5ug/mL Blasticidin. *Gaussia* and *Cypridina* Luciferase constructs were obtained from New England BioLabs (Ipswich, MA). The hMLuc coding region was subcloned into the GLuc expression vector, replacing GLuc, and confirmed by restriction mapping.

Reporter Comparisons. HEK293 cells were transiently transfected with pCMV-hMLuc, pCMV-GLuc, or pCMV-CLuc and RpF-GFP. Forty-eight hours after transfection GFP expression was quantified by fluorimeter and luciferase activity was measured from conditioned medium and cell lysates. Transfection efficiency was normalized by GFP.

Cellular dilution studies: HEK293 cells were transiently transfected with pCMV-hMLuc, pCMV-GLuc, or pCMV-CLuc and RpF-GFP. After 24 hours these cells, and the stably transfected HCT-116-hMLuc cells, were counted and serial dilutions plated in 96 well plates. After 24 hours 100 μl of the conditioned media was transferred to a Corning multiwall plate for MLuc quantification. Correlation coefficients were determined by linear regression analysis.
