# Supplementary material for: A Real Time Metridia Luciferase Based Non-Invasive Reporter Assay of Mammalian Cell Viability and Cytotoxicity via the β-actin Promoter and Enhancer
Source: PLoS One. 2012 May 9;7(5):e36535. doi: 10.1371/journal.pone.0036535 (PMC3348918; doi:10.1371/journal.pone.0036535)
Supplement: Figure S1 — Linear quantification of MLuc Activity with cell number. Serially diluted samples of (A) stable HCT116-hMLuc and (B) transiently transfected HEK293 cells (pDonor-hβactin-hMLuc) were plated in 96 well plates (n = 5). Twenty four hours after plating, 100 microliters of conditioned media was taken from each well and evaluated for MLuc activity. Error bars represent standard error of the mean. The X-axis represents plated cell number and the Y-axis represents MLuc Activity (Relative Light Units or RLU) for each sample. Linear regression analysis indicates an R2 value of 0.998 for (A) HCT116-hMLuc cells and 0.987 for (B) HEK293 cells. (PDF) [file pone.0036535.s002.pdf]

A

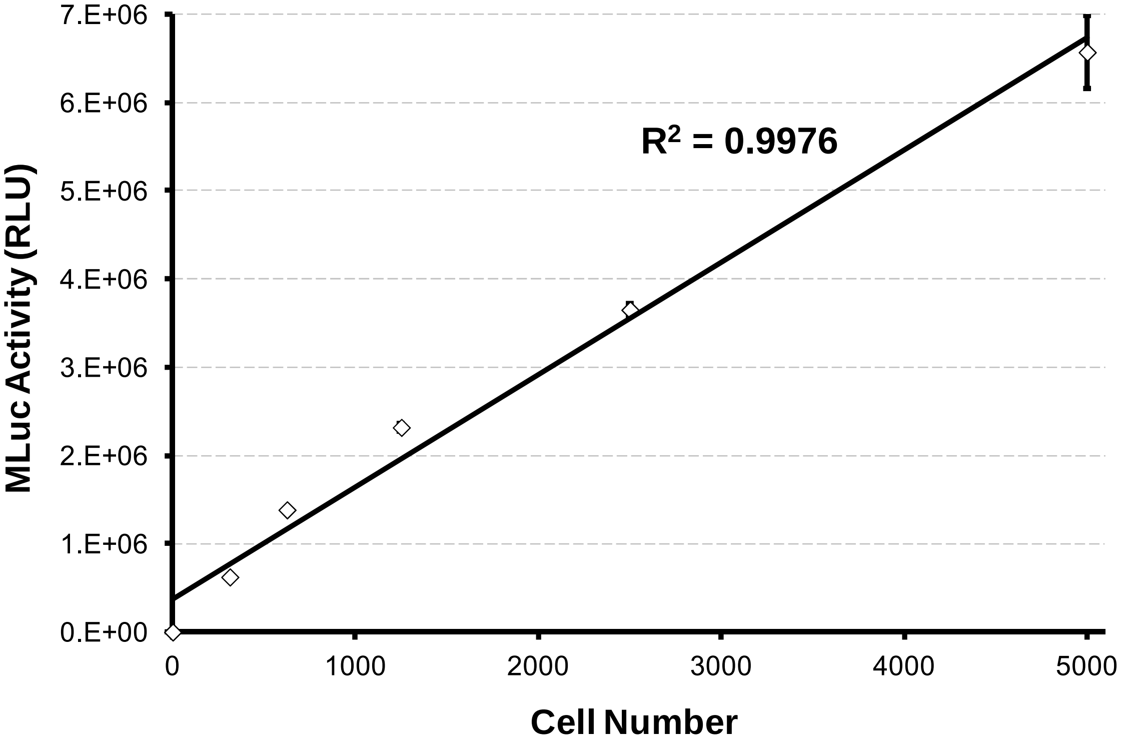

B

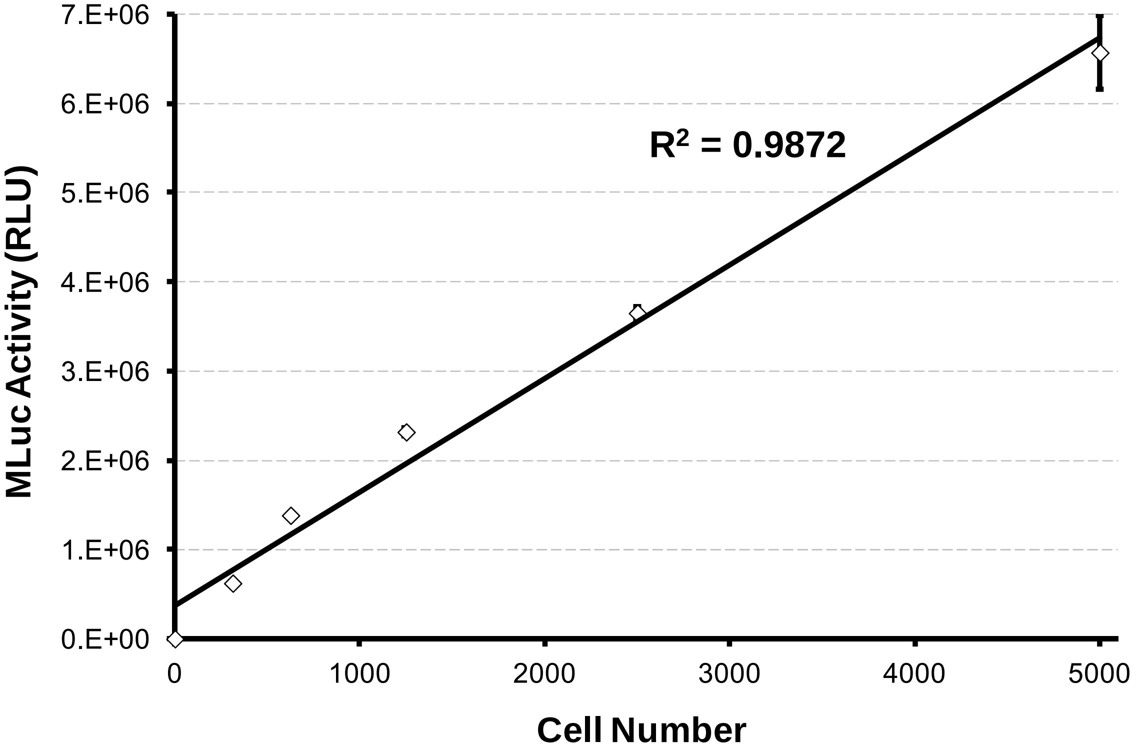

Supplementary Figure S1. Linear quantification of MLuc Activity with cell number. Serially diluted samples of (A) stable HCT116-hMLuc and (B) transiently transfected HEK293 cells (pDonor-hβactin-hMLuc) were plated in 96 well plates (n=5). Twenty four hours after plating, 100 microliters of conditioned media was taken from each well and evaluated for MLuc activity. Error bars represent standard error of the mean. The X-axis represents plated cell number and the Y-axis represents MLuc Activity (Relative Light Units or RLU) for each sample. Linear regression analysis indicates an R^2 value of 0.998 for (A) HCT116-hMLuc cells and 0.987 for (B) HEK293 cells.
