# Supplementary material for: A Real Time Metridia Luciferase Based Non-Invasive Reporter Assay of Mammalian Cell Viability and Cytotoxicity via the β-actin Promoter and Enhancer
Source: PLoS One. 2012 May 9;7(5):e36535. doi: 10.1371/journal.pone.0036535 (PMC3348918; doi:10.1371/journal.pone.0036535)
Supplement: Figure S2 — Comparison of secreted luciferase constructs. HEK293 cells were transiently transfected with RpF-GFP and pCDNA-3.1-hMLuc, pCDNA-3.1-GLuc, or pCDNA-3.1-CLuc. Forty eight hours after transfection luciferase activity was measured from conditioned medium and cell lysates and RLU activity normalized to GFP expression. the (A) percent activity of cell fractions as well as the (B) total activity as reported as Relative Light Units (RLU) are plotted. Error bars represent standard error of the mean. N = 8. (PDF) [file pone.0036535.s003.pdf]

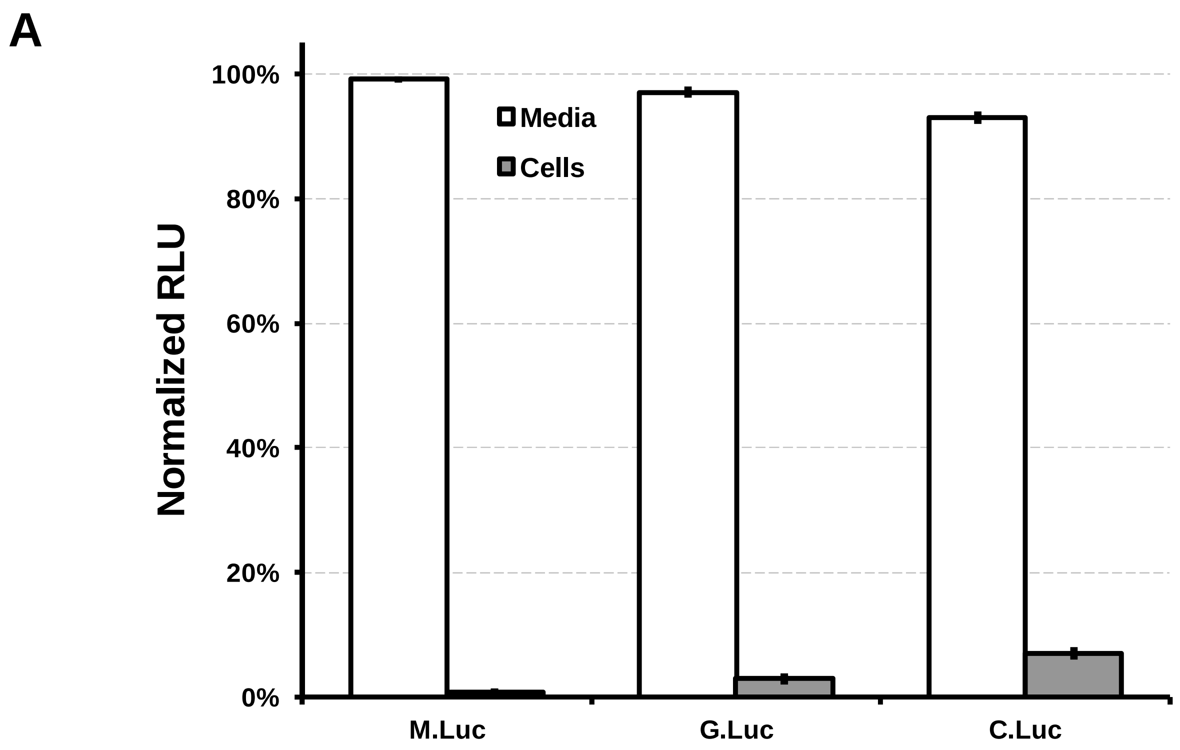

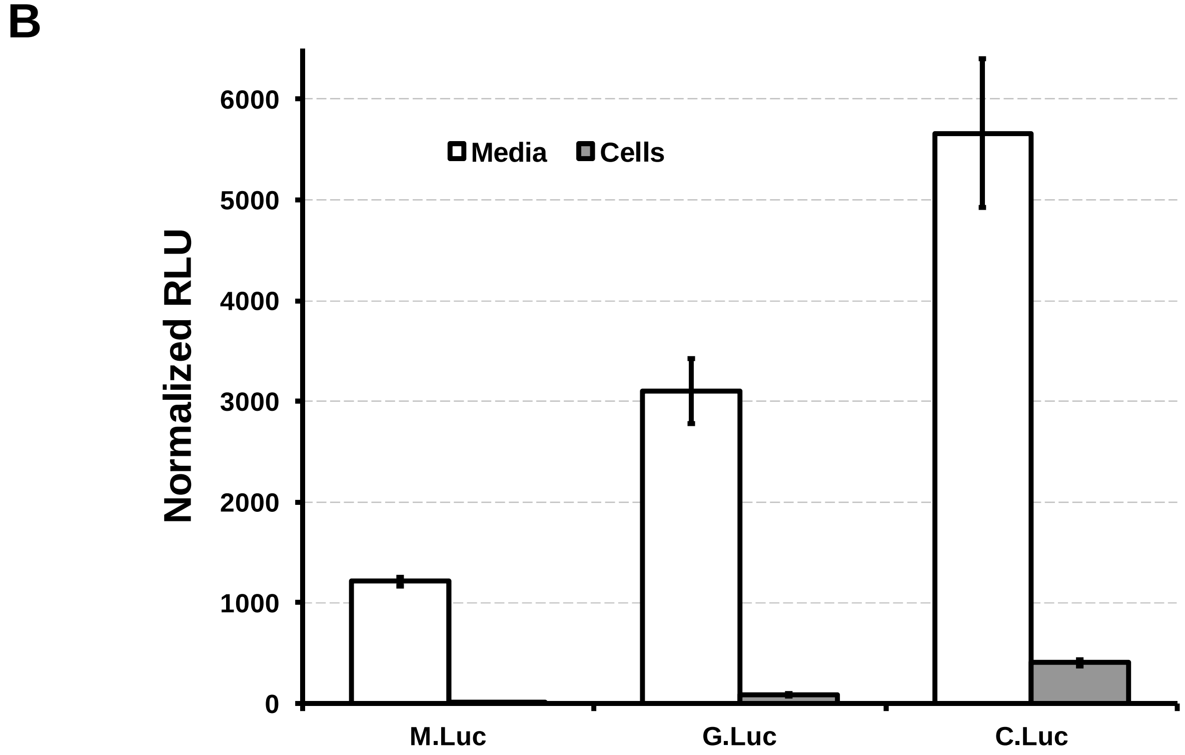

Supplementary Figure S2. Comparison of secreted luciferase constructs. HEK293 cells were transiently transfected with RpF-GFP and pCDNA-3.1-hMLuc, pCDNA-3.1-GLuc, or pCDNA-3.1-CLuc . Forty eight hours after transfection luciferase activity was measured from conditioned medium and cell lysates and RLU activity normalized to GFP expression. Both the (A) percent activity of cell fractions as well as the (B) total activity as reported as Relative Light Units (RLU) are plotted. Error bars represent standard error of the mean. N=8.
